# Procalcitonin decrease predicts survival and recovery from dialysis at 28 days in patients with sepsis-induced acute kidney injury receiving continuous renal replacement therapy

**DOI:** 10.1371/journal.pone.0279561

**Published:** 2022-12-27

**Authors:** Il Young Kim, Suji Kim, Byung Min Ye, Min Jeong Kim, Seo Rin Kim, Dong Won Lee, Soo Bong Lee

**Affiliations:** 1 Department of Internal Medicine, Pusan National University School of Medicine, Yangsan, Republic of Korea; 2 Research Institute for Convergence of Biomedical Science and Technology, Pusan National University Yangsan Hospital, Yangsan, Republic of Korea; Rutgers New Jersey Medical School, UNITED STATES

## Abstract

Procalcitonin (PCT) is a biomarker for diagnosing infections and guiding antibiotic therapy. In this study, we investigated whether PCT can predict survival and recovery at 28 days in critically ill patients with sepsis-induced acute kidney injury (SIAKI) receiving continuous renal replacement therapy (CRRT). We examined 649 patients with SIAKI who underwent CRRT in our intensive care unit. In a multivariable Cox regression analysis, a single PCT level at CRRT initiation was not associated with survival in all patients. However, the higher % PCT decrease over 72 hours after CRRT initiation was independently associated with the higher chance of 28-day survival (per 10% decrease, hazard ratio [HR] for mortality: 0.87, 95% confidence interval [CI]: 0.85–0.89; *P* < 0.001). Among the survivors, the % PCT decrease over 72 hours after CRRT initiation, not a single PCT level at CRRT initiation, was independently associated with recovery from dialysis (per 10% decrease, HR for renal recovery: 1.28, 95% CI:1.21–1.36; *P* < 0.001). This study demonstrated that the higher % PCT decrease was independently associated with the higher chance of survival and recovery from dialysis at 28 days in critically ill patients with SIAKI receiving CRRT. Thus, a decrease in the PCT level, not a single PCT level at CRRT initiation, could be a valuable tool for predicting prognosis in these patients.

## Introduction

Acute kidney injury (AKI) is a common and serious complication that occurs in more than 50% of critically ill patients [1]. Mortality among critically ill patients with AKI has been reported to be more than 50% and as high as 80% in patients requiring renal replacement therapy (RRT) [2–4]. Sepsis is the leading cause of AKI in patients admitted to the intensive care unit (ICU), and accounts for approximately 50% of all the AKI cases [3, 5]. Continuous renal replacement therapy (CRRT) is the most common dialysis therapy for critically ill patients who are hemodynamically unstable [6]. Despite the improvements in intensive care, mortality in critically ill patients with sepsis-induced acute kidney injury (SIAKI) receiving CRRT remains up to 50% [7]. Additionally, 25% of these patients remain dialysis dependent upon hospital discharge [6].

Risk stratification tools such as disease severity scores and various types of biomarkers have been incorporated into the management of patients with AKI, since the estimated probabilities of survival and recovery from dialysis may provide important information for clinical decision making such as providing realistic prognostic information to patients and their families, optimizing limited resources, and discussing goal-of-treatment [8, 9]. However, it has been suggested that general prognostic tools lack predictive accuracy or exhibit significant variability when used in patients with AKI [8]. In critically ill patients, the disease severity scores such as Acute Physiology and Chronic Health Inquiry II (APACHE II) and Sequential Organ Failure Assessment (SOFA) reportedly perform poorly in patients with AKI receiving CRRT [6]. Thus, disease-specific risk stratification tools have been developed for critically ill patients with AKI [8]; however, the data regarding the risk of survival and recovery from dialysis in critically ill patients with SIAKI receiving CRRT are scarce.

Procalcitonin (PCT) is a pro-hormone of the calcium metabolism regulator calcitonin, and is synthesized by the parafollicular C cells of the thyroid [10, 11]. PCT is a biomarker of systemic bacterial infection and sepsis, because it is synthesized in numerous extrathyroidal tissues in response to lipopolysaccharides and bacteria-induced cytokines [11]. Traditionally, PCT has been recognized as a biomarker for differentiating between bacterial and non-bacterial infections and for guiding antibiotic treatment [12]. Recently, PCT has been reported to be associated with the severity of systemic infections in sepsis [13], and is considered to have diagnostic and prognostic value in patients with sepsis [10]. Indeed, a recent meta-analysis demonstrated that elevated PCT concentrations and non-clearance of PCT are strongly associated with all-cause mortality in patients [14]. Concerning the relationship between PCT and AKI, a recent systemic review suggested that the PCT level is a biomarker for predicting AKI development in various clinical settings regardless of infection [15].

Taken together, although PCT is reported to be a predictor of survival and AKI development in septic patients, the association between PCT levels and survival or recovery from dialysis in patients with SIAKI receiving CRRT remains unknown. In this study, we aimed to investigate whether a single PCT level at CRRT initiation or a decrease in the PCT level over 72 hours after CRRT initiation had a prognostic value in predicting the survival and recovery from dialysis at 28 days in patients with SIAKI receiving CRRT.

## Materials and methods

### Study population

**Fig 1:**
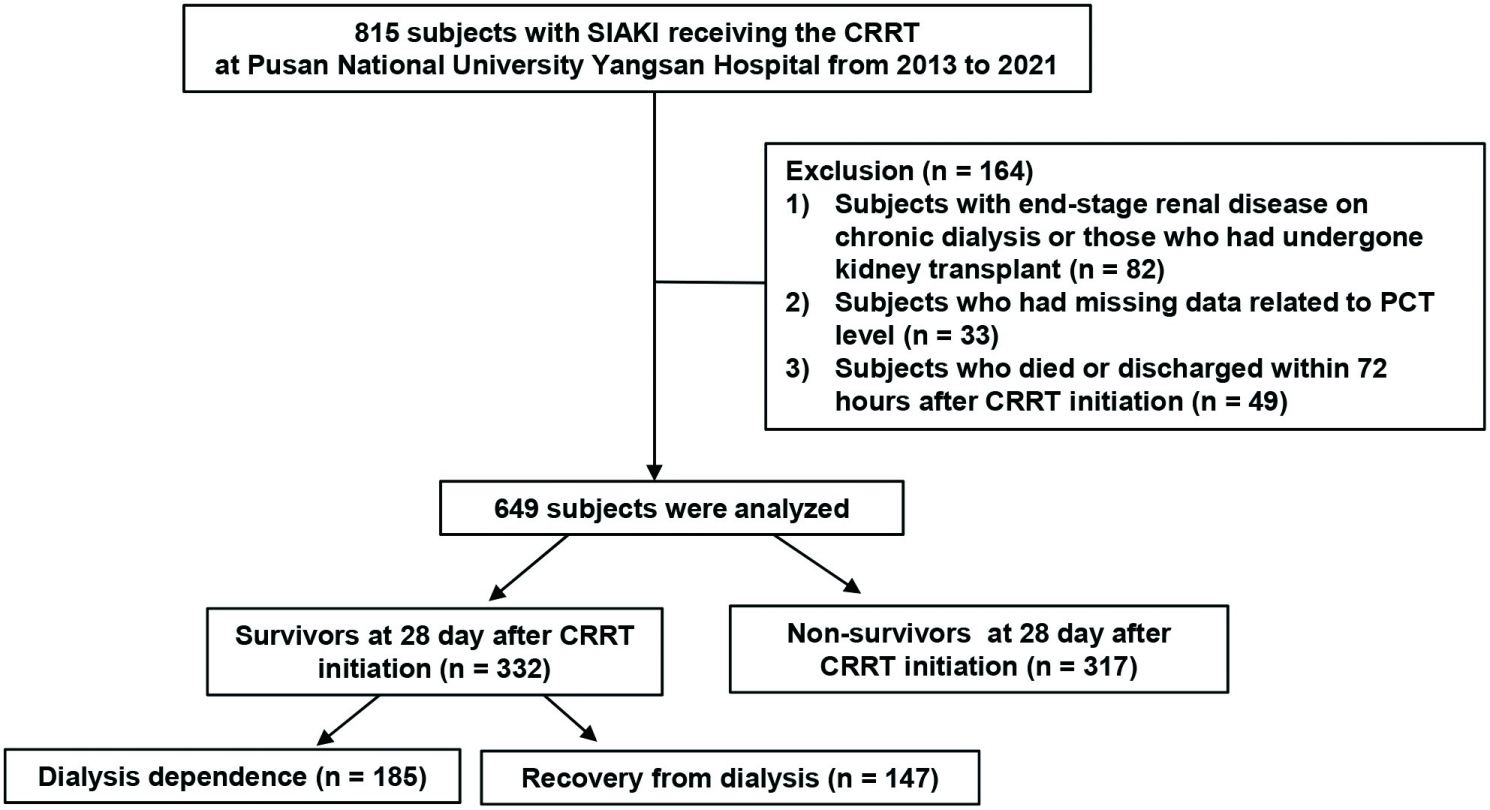
Flow diagram for patient enrollment. CRRT, continuous renal replacement therapy; ICU, intensive care unit; PCT, procalcitonin; SIAKI, sepsis-induced acute kidney injury.

We conducted a single-center retrospective cohort study of patients admitted to the ICU at Pusan National University Yangsan Hospital between 2013 and 2021. We initially recruited a total of 815 adult patients (age ≥ 18 years) with sepsis and AKI who underwent CRRT. The exclusion criteria were as follows: (a) end-stage renal disease on chronic dialysis or history of kidney transplantation, (b) missing data related to the PCT level, and (c) death or discharge within 72 hours of CRRT initiation. Finally, 649 patients were examined (Fig 1). All research and data collection processes were conducted in accordance with the Declaration of Helsinki and the current ethical guidelines. The study protocol was approved by the hospital’s Institutional Review Board (IRB) (Pusan National University Yangsan Hospital Review Board, IRB No. 05-2022-091). The need for informed consent was waived by the IRB because of the retrospective nature of the analysis, which used anonymized information contained in medical charts and records.

### Data collection

We reviewed the electronic medical records and collected the demographic and clinical data at ICU admission, including age, sex, body weight, comorbidities (chronic kidney disease [CKD], hypertension, diabetes, chronic obstructive pulmonary disease [COPD], liver cirrhosis, congestive heart failure, solid cancer, and hematologic cancer), infection source (respiratory, gastrointestinal, urinary tract, and soft tissue), severity of illness (SOFA score, APACHE II, vasopressor use, and ventilator dependency), mean arterial pressure, fever, heart rate, and oliguria (<0.5 ml/kg/h for 6 hours before CRRT initiation). Blood examinations were performed upon CRRT initiation, which included the analysis of creatinine, blood urea nitrogen, potassium, sodium, leukocyte count, hemoglobin, platelet count, total bilirubin, albumin, prothrombin time (international normalized ratio) (PT [INR]), C-reactive protein, and lactate levels. The time intervals between AKI diagnosis and CRRT initiation, CRRT duration, and the prescribed CRRT dose were investigated.

The initial level of PCT was measured on the day of CRRT initiation (PCT0). For patients with more than one available follow-up PCT measurement at 72 ± 12 hours after CRRT initiation, the measurement closest to 72 hours was used for the analyses (PCT72h). Based on previous studies that investigated the dynamic change in PCT levels in patients with sepsis, we chose 72 hours as the time interval for dynamic changes in PCT levels [11, 16, 17]. A change in the PCT levels over 72 hours after CRRT initiation was expressed as a % PCT decrease. The % PCT decrease was calculated using the following formula: % PCT decrease = [(PCT0—PCT72h)/PCT0] × 100. The % PCT decrease was observed to be positive with decreasing concentration and negative with increasing concentration. The PCT levels were measured using the Elecsys BRAHMS PCT assay (Roche Diagnostics, Mannheim, Germany; the inter-assay and intra-assay coefficients of variation were 0.9–1.3% and 2.2–5.6%) on a Roche Cobas c501 instrument between 2013 and 2018 and with BRAHMS PCT-sensitive Kryptor (Thermo Fisher Scientific, Hennigsdorf, Germany; the inter-assay and intra-assay coefficients of variation were 2.9–5.8% and 3.5–6.5%) on the Kryptor Compact Plus instrument between 2019 and 2021.

### CRRT protocol

The primary indications for CRRT initiation in patients with sepsis and AKI were as follows: a medically intractable volume overload, electrolyte imbalance, metabolic acidosis, oliguria with progressive azotemia, and hemodynamic instability. Decisions regarding when to initiate or terminate CRRT as well as the CRRT setting (target clearance, blood flow, dialysate/replacement fluid rate, and anticoagulation) were made through consultations and discussions with the attending nephrologists or intensivists. All the patients received continuous venovenous hemodiafiltration using Prisma or Prismaflex (Baxter, IL, USA) with an AN-69 polyacrylonitrile membrane dialyzer. A venous catheter for CRRT was inserted into the internal jugular or femoral veins. CRRT was initiated with the blood flow, which was gradually increased to 150 ml/min. A CRRT dose of 35–40 ml/kg/h was prescribed to ensure a delivered CRRT dose of ≥ 35 ml/kg/h.

### Definition and study outcome

Sepsis was defined according to the American College of Chest Physicians/Society of Critical Care Medicine consensus conference criteria [18]. Sepsis was diagnosed if patients had a proven or strongly suspected bacterial infection and had at least two of the following systemic inflammatory response syndrome criteria: body temperature > 38 °C or < 36 °C, heart rate > 90 bpm, respiratory rate > 20 breaths/min, PaCO_2_ < 32 mmHg or use of mechanical ventilation, white cell count > 12,000/mm^3^ or < 4,000/mm^3^, or immature neutrophils > 10%). AKI diagnosis was based on Kidney Disease: Improving Global Outcomes (KDIGO) clinical practice guidelines for AKI i.e., increase in serum creatinine ≥ 0.3 mg/dL within 48 h, increase in serum creatinine ≥ 1.5-times the baseline value, or urine volume *<* 0.5 ml/kg/h for 6 hours) [19]. The main outcome was survival within 28 days after CRRT initiation. Another outcome was recovery from dialysis among the survivors, which was defined as the status of being free from any form of RRT, including CRRT and intermittent hemodialysis within 28 days after CRRT initiation.

### Statistical analysis

Continuous variables were expressed as medians with interquartile ranges and were compared using the Mann–Whitney test. Categorical variables were expressed as numbers with percentages and compared using the chi-square test. To determine the independent predictors for survival and recovery from dialysis within 28 days after CRRT initiation, univariable and multivariable Cox proportional hazards analyses were used, and the results were presented as hazard ratios (HR) and 95% confidence intervals (CIs). Significant variables were identified through univariable analysis (*P* < 0.1), and clinically important variables were considered in the multivariable analysis. Of the significant variables in the univariable analysis, those included in the SOFA or APACHE II scores i.e., mean arterial pressure, platelet count, pH, and serum creatinine were excluded from the multivariable analysis to avoid a redundant analysis. Instead, the SOFA and APACHE II scores for these variables were considered in the final multivariable analysis.

A receiver operating characteristic (ROC) curve analysis was used to assess the area under the curve (AUC), and the Youden index was used to determine the best cut-off value of % PCT decrease for predicting survival and recovery from dialysis within 28 days after CRRT initiation. We also performed a Kaplan–Meier analysis and log-rank test to compare the survival and recovery from dialysis between the groups, which was stratified by the best cut-off value of % PCT decrease for predicting survival and recovery from dialysis. The statistical significance was set at *P* < 0.05. All analyses were performed using SPSS (version 26.0; SPSS, Inc., Chicago, IL, USA) and MedCalc Statistical Software version 19.4.1 (MedCalc Software, Ostend, Belgium).

## Results

### Baseline characteristics stratified by mortality after CRRT initiation

Of the 649 patients, 317 patients died within 28 days of CRRT initiation. The baseline characteristics of the study population stratified by the 28-day survival after CRRT initiation are presented in Table 1. Regarding demographics, the non-survivors were older than the survivors. There were no significant differences in the sex or body weight between the survivors and non-survivors. In terms of comorbid diseases, the non-survivors had a higher prevalence of COPD, liver cirrhosis, congestive heart failure, and solid cancer than the survivors. There were no significant differences in the infection sources (respiratory, gastrointestinal, urinary tract, and soft tissue) between survivors and non-survivors. Regarding the severity of illness at CRRT initiation, non-survivors had higher SOFA and APACHE II scores and higher prevalence of vasopressor use and ventilator dependency as compared to the survivors. At CRRT initiation, non-survivors had lower mean arterial pressure, platelet count, and pH as compared to the survivors. Non-survivors were also likely to have a higher prevalence of oliguria, along with elevated prothrombin time and lactate levels than non-survivors. The interval between AKI diagnosis and CRRT initiation was significantly higher in non-survivors than in survivors. There were no significant differences in the prescribed CRRT doses between the two groups. In terms of a single PCT level, non-survivors had a higher PCT concentration at CRRT initiation (14.2 ng/ml [7.3–22.8] vs. 10.4 ng/ml [4.8–17.5], *P* < 0.001) and at 72 hours after CRRT initiation (13.5 ng/ml [6.4–24.9] vs. 3.6 ng/ml [0.8–9.8], *P* < 0.001) than the survivors. In terms of the % PCT decrease, the survivors demonstrated higher levels of % PCT decrease than the non-survivors (63.0% [4.0–90.0] vs. -19.0% [-16.0–15.3], *P* < 0.001).

**Table 1:** Baseline characteristics stratified by survival at 28 days after CRRT initiation (n = 649).

Variables	Survivor (n = 332)	Non-survivor (n = 317)	*P*
Demographics			
Age (years)	69 (58–77)	72 (62–80)	0.006
Sex, male	202 (60.8%)	194 (61.2%)	0.926
Baseline body weight (kg)	65 (56–74)	65 (57–75)	0.784
Comorbid disease, n (%)			
Chronic kidney disease	70 (21.1%)	82 (25.9%)	0.150
Hypertension	172 (51.8%)	179 (56.5%)	0.234
Diabetes mellitus	117 (35.2%)	122 (38.5%)	0.392
COPD	31 (9.3%)	58 (18.3%)	0.001
Liver cirrhosis	37 (11.1%)	70 (22.1%)	<0.001
Congestive heart failure	36 (10.8%)	62 (19.6%)	0.002
Solid cancer	55 (16.6%)	74 (23.3%)	0.031
Hematologic cancer	20 (6.0%)	21 (6.6%)	0.753
Infection source, n (%)			
Respiratory	118 (35.5%)	114 (36.0%)	0.911
Gastrointestinal	100 (30.1%)	109 (34.4%)	0.245
Urinary tract	94 (28.3%)	74 (23.3%)	0.149
Soft tissue	9 (2.7%)	13 (4.1%)	0.328
Unknown	11 (3.3%)	7 (2.2%)	0.391
Severity of illness at CRRT initiation			
SOFA score	11 (6–15)	17 (13–20)	<0.001
APACHE II score	24 (21–30)	32 (27–35)	<0.001
Vasopressors use, n (%)	112 (33.7%)	219 (69.1%)	<0.001
Ventilator dependency, n (%)	97 (29.2%)	236 (74.4%)	<0.001
Findings at CRRT initiation			
Mean arterial pressure (mmHg)	80 (70–93)	66 (55–82)	<0.001
Fever or hypothermia, n (%)	123 (37.0%)	122 (38.5%)	0.706
Heart rate (beat/minute)	97 (79–115)	99 (82–117)	0.441
Oliguria for 6 hours before CRRT (< 0.5 ml/kg/h), n (%)	85 (25.6%)	214 (67.5%)	<0.001
Creatinine (mg/dl)	2.7 (2.3–3.4)	2.7 (2.4–4.0)	0.092
Blood urea nitrogen (mg/dl)	50.2 (41.6–68.8)	51.6 (40.9–70.1)	0.789
Sodium (meq/l)	136 (130–141)	135 (130–141)	0.497
Potassium (meq/l)	4.6 (4.0–5.2)	4.7 (4.0–5.3)	0.221
Leukocyte count (1000/mm^3^)	13.1 (10.1–18.8)	13.4 (9.7–18.9)	0.688
Hemoglobin (g/dl)	9.0 (8.0–10.9)	9.5 (7.7–11.1)	0.649
Platelet count (1000/mm^3^)	115 (71–159)	79 (53–118)	<0.001
Total bilirubin (mg/dl)	1.7 (0.8–3.9)	1.9 (0.5–4.2)	0.646
Albumin (g/dl)	2.7 (2.3–3.1)	2.6 (2.3–3.0)	0.255
PT(INR)	1.6 (1.3–1.9)	1.7 (1.5–2.0)	0.003
CRP (mg/dl)	11.3 (6.7–25.5)	14.3 (7.7–26.4)	0.087
pH	7.27 (7.23–7.35)	7.22 (7.16–7.31)	<0.001
Lactate (mmol/l)	2.8 (1.9–6.8)	7.4 (4.3–11.5)	<0.001
CRRT			
Interval time from AKI diagnosis to CRRT initiation (days)	0.3 (0.3–1.4)	1.6 (0.8–2.4)	<0.001
Prescribed CRRT dose (ml/kg/h)	37.6 (35.4–39.9)	37.5 (35.0–40.0)	0.514
PCT concentration (ng/ml)			
At CRRT initiation (PCT0)	10.4 (4.8–17.5)	14.2 (7.3–22.8)	<0.001
At 72 ± 12 hours after CRRT initiation (PCT72h)	3.6 (0.8–9.8)	13.5 (6.4–24.9)	<0.001
% PCT decrease (%)^a^	63.0 (4.0–90.0)	-19.0 (-46.0–15.3)	<0.001

Values are expressed as median (interquartile range) or percentage (%). AKI, acute kidney injury; APACHE II, Acute Physiology and Chronic Health Evaluation II; COPD, chronic obstructive pulmonary disease; CRP, C-reactive protein; CRRT, continuous renal replacement therapy; PCT, procalcitonin; PT (INR), prothrombin time (international normalized ratio); SOFA, Sequential Organ Failure Assessment.

^a^% PCT decrease = [(PCT0-PCT72h)/PCT0] × 100. The % PCT decrease was positive with decreasing concentration and negative with increasing concentration.

### Baseline characteristics stratified by recovery from dialysis after CRRT initiation among survivors

Among the survivors (n = 332), 147 patients were free from dialysis within 28 days of CRRT initiation. The baseline characteristics stratified by recovery from dialysis within 28 days of CRRT initiation are presented in Table 2. The patients who recovered from dialysis had a lower prevalence of CKD than dialysis-dependent patients. In terms of the severity of illness at CRRT initiation, the patients who recovered from dialysis had lower SOFA and APACHE II scores and a lower prevalence of vasopressor use and ventilator dependency than dialysis-dependent patients. Regarding the findings at CRRT initiation, the patients who recovered from dialysis had a lower prevalence of oliguria and lower levels of serum creatinine, prothrombin time, CRP, and lactate than dialysis-dependent patients. Concerning a single PCT level, the patients who recovered from dialysis had a lower PCT concentration at both CRRT initiation (8.8 ng/ml [3.5–17.0] vs. 11.3 ng/ml [6.6–17.5], *P* < 0.001) and 72 hours after CRRT initiation (1.0 ng/ml [0.4–3.0] vs. 7.2 ng/ml [3.3–13.5], *P* < 0.001) as compared to the dialysis-dependent patients. In terms of the % PCT decrease, patients who recovered from dialysis had higher levels of % PCT decrease than dialysis-dependent patients (87.0% [67.3–94.0] vs. 17.0 [-11.3–66.0], *P* < 0.001).

**Table 2:** Baseline characteristics stratified by recovery from dialysis at 28 days after CRRT initiation among the survivors (n = 332).

Variables	Dialysis dependence (n = 185)	Recovery from dialysis (n = 147)	*P*
Demographics			
Age (years)	69 (59–79)	68 (57–75)	0.097
Sex, male	106 (57.3%)	96 (65.3%)	0.183
Baseline body weight (kg)	64 (56–74)	65 (56–75)	0.746
Comorbid disease, n (%)			
Chronic kidney disease	61 (33.0%)	9 (6.1%)	<0.001
Hypertension	99 (53.5%)	73 (49.7%)	0.485
Diabetes mellitus	68 (36.8%)	49 (33.3%)	0.517
COPD	22 (11.9%)	9 (6.1%)	0.073
Liver cirrhosis	32 (11.9%)	15 (10.2%)	0.627
Congestive heart failure	24 (13.0%)	12 (8.2%)	0.162
Solid cancer	32 (17.3%)	23 (15.6%)	0.688
Hematologic cancer	12 (6.5%)	8 (5.4%)	0.691
Infection source, n (%)			
Respiratory	71 (38.4%)	47 (32.0%)	0.226
Gastrointestinal	56 (30.3%)	44 (29.9%)	0.947
Urinary tract	46 (24.9%)	48 (32.7%)	0.118
Soft tissue	7 (3.8%)	2 (1.4%)	0.177
Unknown	5 (2.7%)	6 (4.1%)	0.486
Severity of illness at CRRT initiation			
SOFA score	13 (9–16)	8 (3–12)	<0.001
APACHE II score	26 (22–32)	23 (20–27)	<0.001
Vasopressors use, n (%)	78 (42.2%)	34 (23.1%)	<0.001
Ventilator dependency, n (%)	69 (37.3%)	28 (19.0%)	<0.001
Findings at CRRT initiation			
Mean arterial pressure (mmHg)	79 (69–93)	86 (70–93)	0.319
Fever or hypothermia, n (%)	69 (37.3%)	54 (36.7%)	0.916
Heart rate (beat/minute)	79 (69–93)	86 (70–93)	0.319
Oliguria for 6 hours before CRRT (< 0.5 ml/kg/h), n (%)	74 (40.0%)	15 (10.2%)	<0.001
Creatinine (mg/dl)	2.7 (2.5–3.4)	2.5 (2.2–3.3)	<0.001
Blood urea nitrogen (mg/dl)	51.3 (41.2–68.5)	49.8 (42.8–69.3)	0.705
Sodium (meq/l)	136 (130–141)	135 (130–141)	0.700
Potassium (meq/l)	4.5 (4.0–5.2)	4.6 (4.1–5.1)	0.537
Leukocyte count (1000/mm^3^)	12.6 (9.5–18.6)	14.3 (10.6–19.6)	0.132
Hemoglobin (g/dl)	8.9 (7.9–10.7)	9.1 (7.7–11.1)	0.590
Platelet count (1000/mm^3^)	115 (67–161)	122 (75–157)	0.782
Total bilirubin (mg/dl)	1.8 (0.8–4.2)	1.7 (0.2–3.4)	0.052
Albumin (g/dl)	2.7 (2.2–3.1)	2.7 (2.4–3.1)	0.639
PT(INR)	1.6 (1.4–2.0)	1.6 (1.3–1.9)	0.040
CRP (mg/dl)	13.3 (7.5–26.5)	9.9 (5.7–24.9)	0.045
pH	7.27 (7.23–7.33)	7.28 (7.20–7.37)	0.372
Lactate (mmol/l)	3.3 (1.9–7.1)	2.5 (1.8–6.2)	0.028
CRRT			
Interval time from AKI diagnosis to CRRT initiation (days)	0.4 (0.3–1.4)	0.3 (0.3–1.2)	0.123
Prescribed CRRT dose (ml/kg/h)	37.5 (35.2–39.9)	37.7 (35.6–39.9)	0.315
PCT concentration (ng/ml)			
At CRRT initiation (PCT0)	11.3 (6.6–17.5)	8.8 (3.5–17.0)	<0.001
At 72 ± 12 hours after CRRT initiation (PCT72h)	7.2 (3.3–13.5)	1.0 (0.4–3.0)	<0.001
% PCT decrease (%)^a^	17.0 (-11.3–66.0)	87.0 (67.3–94.0)	<0.001

Values are expressed as median (interquartile range) or percentage (%). AKI, acute kidney injury; APACHE II, Acute Physiology and Chronic Health Evaluation II; COPD, chronic obstructive pulmonary disease; CRP, C-reactive protein; CRRT, continuous renal replacement therapy; PCT, procalcitonin; PT (INR), prothrombin time (international normalized ratio); SOFA, Sequential Organ Failure Assessment.

^a^% PCT decrease = [(PCT0-PCT72h)/PCT0] × 100. The % PCT decrease was positive with decreasing concentration and negative with increasing concentration.

### Association between % PCT decrease and survival

Table 3 shows the variables found to be associated with the 28-day survival in the study subjects. In the univariable Cox regression analysis, both PCT concentration at CRRT initiation and % PCT decrease over 72 hours after CRRT initiation were predictors of 28-day survival. Furthermore, age, COPD, liver cirrhosis, congestive heart failure, SOFA score, APACHE II score, vasopressor use, ventilator use, mean arterial pressure, oliguria, serum creatinine, platelet count, PT (INR), pH, lactate level, and time interval between AKI diagnosis and CRRT initiation were significant predictors of 28-day survival. In the multivariable Cox regression analysis, the % PCT decrease over 72 hours after CRRT initiation was an independent predictor of 28-day survival (per 10% decrease, HR for mortality: 0.91, 95% CI: 0.89–0.93; *P* < 0.001). However, a single PCT level at CRRT initiation was not a predictor of the 28-day survival. In addition, congestive heart failure (HR for mortality: 1.40, 95% CI: 1.04–1.87; *P* = 0.026), SOFA score (per 1 point increase, HR for mortality: 1.10, 95% CI: 1.07–1.13; *P* < 0.001), APACHE II score (per 1 point increase; HR for mortality: 1.04, 95% CI: 1.02–1.07; *P* < 0.001), oliguria (HR for mortality: 1.56, 95% CI: 1.21–2.03, *P* = 0.001), lactate (per 1.0 mmol/L increase, HR for mortality: 1.05, 95% CI: 1.02–1.08; *P* < 0.001), and interval time from AKI diagnosis to CRRT initiation (per 1 day increase, HR for mortality: 1.11, 95% CI: 1.00–1.24; *P* = 0.043) were independent predictors of 28-day survival.

**Table 3:** Univariable and multivariable Cox regression analyses for survival within 28 days after CRRT initiation (n = 649).

Variables	Univariable		Multivariable	
	HR (95% CI) for mortality	*P*	HR (95% CI) for mortality	*P*
Demographics				
Age (per 1 year increase)	1.01 (1.00–1.02)	0.005	1.00 (0.99–1.00)	0.283
Sex, male	0.98 (0.78–1.23)	0.850	0.92 (0.72–1.17)	0.476
Baseline body weight (per 1.0 kg increase)	1.00 (0.99–1.01)	0.985		
Comorbid disease				
Chronic kidney disease	1.18 (0.92–1.52)	0.198		
Hypertension	1.11 (0.89–1.38)	0.375		
Diabetes mellitus	1.09 (0.87–1.36)	0.481		
Chronic obstructive pulmonary disease	1.61 (1.21–2.14)	0.001	1.06 (0.79–1.42)	0.707
Liver cirrhosis	1.57 (1.20–2.05)	0.001	1.21 (0.92–1.59)	0.176
Congestive heart failure	1.54 (1.17–2.03)	0.002	1.40 (1.04–1.87)	0.026
Solid cancer	1.30 (1.00–1.68)	0.051	1.20 (0.91–1.57)	0.196
Hematologic cancer	1.10 (0.71–1.71)	0.681		
Infection source				
Respiratory	1.03 (0.82–1.29)	0.810		
Gastrointestinal	1.13 (0.89–1.42)	0.316		
Urinary tract	0.83 (0.64–1.07)	0.151		
Soft tissue	1.41 (0.81–2.46)	0.225		
Unknown	0.72 (0.34–1.52)	0.386		
Severity of illness				
SOFA score (per 1 point increase)	1.15 (1.12–1.18)	<0.001	1.10 (1.07–1.13)	<0.001
APACHE II score (per 1 point increase)	1.11 (1.09–1.13)	<0.001	1.04 (1.02–1.07)	<0.001
Vasopressors use	2.84 (2.24–3.61)	<0.001		
Ventilator dependency	4.17 (3.24–5.38)	<0.001		
Findings at CRRT initiation				
Mean arterial pressure (per 1.0 mmHg increase)	0.97 (0.96–0.97)	<0.001		
Fever or hypothermia	1.06 (0.85–1.33)	0.611		
Heart rate (per 1.0 beat/minute increase)	1.00 (1.00–1.01)	0.420		
Oliguria for 6 hours before CRRT (<0.5 ml/kg/h)	3.38 (2.67–4.28)	<0.001	1.56 (1.21–2.03)	0.001
Serum creatinine (per 1.0 mg/dl increase)	1.11 (1.02–1.20)	0.019		
Serum blood urea nitrogen (per 1.0 mg/dl increase)	1.00 (1.00–1.00)	0.562		
Sodium (per 1.0 meq/l increase)	1.00 (0.98–1.01)	0.729		
Potassium (per 1.0 meq/l increase)	1.06 (0.95–1.18)	0.333		
Leukocyte count (per 1000/mm^3^ increase)	1.01 (0.99–1.02)	0.421		
Hemoglobin (per 1.0 g/dl increase)	1.00 (0.95–1.06)	0.871		
Platelet count (per 1000/mm^3^ increase)	0.99 (0.99–1.00)	<0.001		
Total bilirubin (per 1.0 mg/dl increase)	1.03 (0.98–1.08)	0.263		
Albumin (per 1.0 g/dl increase)	0.90 (0.73–1.11)	0.310		
PT(INR) (per 1.0 increase)	1.20 (1.01–1.43)	0.038	0.83 (0.67–1.02)	0.073
CRP (per 1.0 mg/dl increase)	1.00 (1.00–1.01)	0.294		
pH (per 0.1 increase)	0.77 (0.71–0.84)	<0.001		
Lactate (per 1.0 mmol/l increase)	1.13 (1.10–1.16)	<0.001	1.05 (1.02–1.08)	<0.001
Interval time from AKI diagnosis to CRRT initiation (per 1 day increase)	1.48 (1.37–1.60)	<0.001	1.11 (1.00–1.24)	0.043
Prescribed CRRT dose (per 1 ml/kg/h increase)	0.99 (0.95–1.03)	0.695		
PCT concentration at CRRT initiation (per 1 ng/ml increase ng/ml)	1.02 (1.01–1.03)	<0.001	1.00 (0.99–1.01)	0.362
% PCT decrease^a^ (per 10% decrease)	0.87 (0.85–0.89)	<0.001	0.91 (0.89–0.93)	<0.001

Values are expressed as median (interquartile range) or percentage (%). AKI, acute kidney injury; APACHE II, Acute Physiology and Chronic Health Evaluation II; COPD, chronic obstructive pulmonary disease; CI, confidence interval; CRP, C-reactive protein; CRRT, continuous renal replacement therapy; HR, hazard ratio; PCT, procalcitonin; PT (INR), prothrombin time (international normalized ratio); SOFA, Sequential Organ Failure Assessment

^a^% PCT decrease = [(PCT0-PCT72h)/PCT0] × 100. The % PCT decrease was positive with decreasing concentration and negative with increasing concentration.

**Fig 2:**
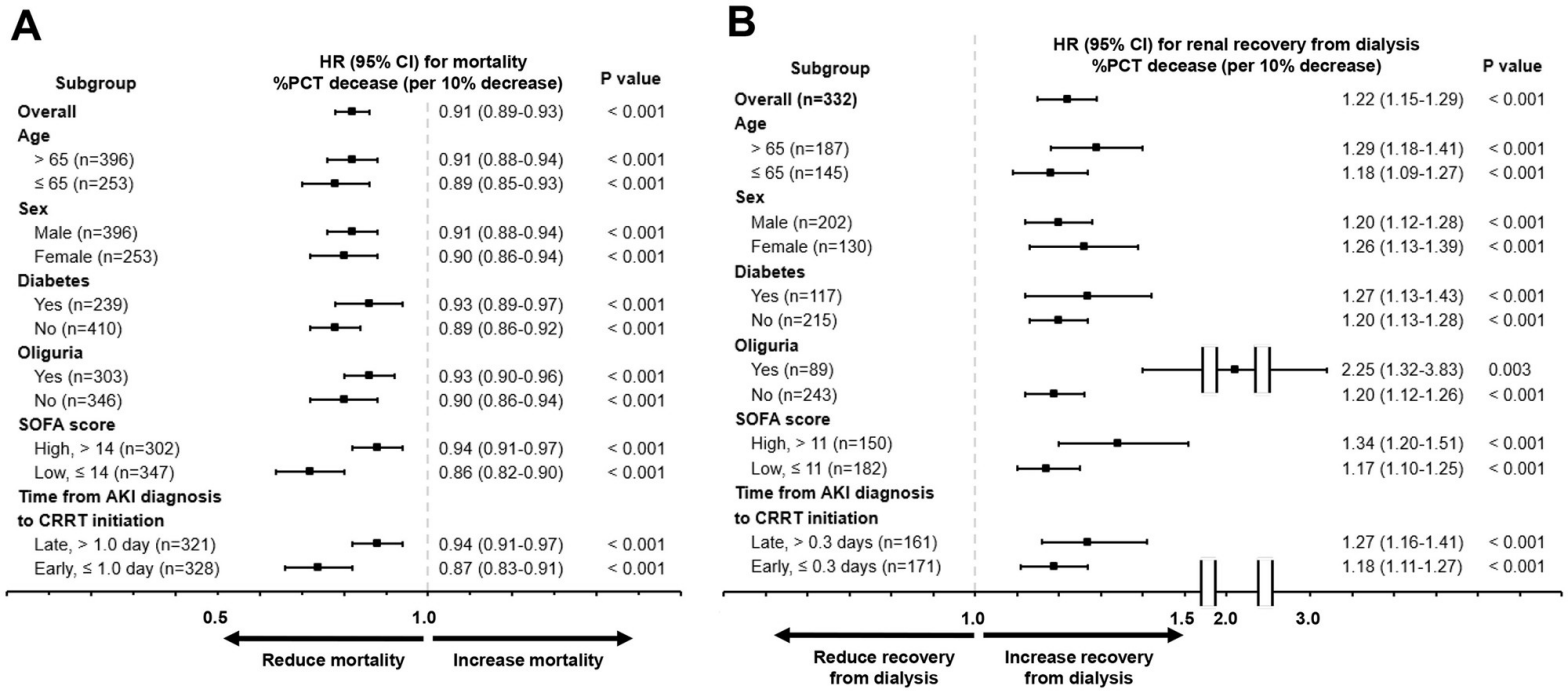
HR plots of % PCT decrease for survival (A) and recovery from dialysis (B) in predefined subgroups. In a multivariable Cox regression analysis, the % PCT decrease was an independent predictor of the 28-day survival in predefined subgroups, including age > 65 or ≤ 65 years, male or female sex, diabetes or no diabetes, oliguria or no oliguria, high SOFA group or low SOFA group, and early CRRT group or late CRRT group. In the survivors, the % PCT decrease was an independent predictor of the recovery from dialysis within 28 days after CRRT initiation across predefined subgroups. CI, confidence interval; CRRT, continuous renal replacement therapy; HR, hazard ratio; PCT, procalcitonin; SOFA, Sequential Organ Failure Assessment.

Next, we investigated the association between % PCT decrease and survival in subgroups that were stratified by age, sex, diabetes, oliguria, SOFA score, and the time from AKI diagnosis to CRRT initiation (Fig 2A). Based on the median SOFA score and the time from AKI diagnosis to CRRT initiation, all the study subjects were categorized into the high SOFA group (> 14 points) and low SOFA group (≤ 14 points) or late CRRT group (> 1.0 day) and early CRRT group (≤ 1.0 day). The multivariable Cox regression analysis revealed that a decrease in the % PCT was an independent predictor of the 28-day survival in predefined subgroups, including age >65 or ≤65 years, male or female sex, diabetes or no diabetes, oliguria or no oliguria, high SOFA group or low SOFA group, and early or late CRRT groups.

### Association between the % PCT decrease and recovery from dialysis

Table 4 shows the variables found to be associated with recovery from dialysis in the survivors. The univariable Cox regression analysis showed that both PCT concentration at CRRT initiation and % PCT decrease over 72 hours after CRRT initiation were predictors of recovery from dialysis. Furthermore, CKD, SOFA and APACHE II scores, vasopressor use, ventilator use, and oliguria were predictors of recovery from dialysis. In the multivariable Cox regression analysis, a % PCT decrease over 72 hours after CRRT initiation was an independent predictor of recovery from dialysis (per 10% decrease, HR for renal recovery: 1.22, 95% CI: 1.15–1.29, *P* < 0.001). However, the PCT level at CRRT initiation alone was not a predictor of recovery from dialysis. In addition, CKD (HR for renal recovery: 0.31, 95% CI: 0.16–0.62; *P* = 0.001), SOFA score (per 1 point increase, HR for renal recovery: 0.95, 95% CI: 0.92–0.98; *P* = 0.002), APACHE II score (per 1 point increase; HR for renal recovery: 0.94, 95% CI: 0.91–0.97; *P* < 0.001), and oliguria (HR for renal recovery: 0.42, 95% CI: 0.24–0.72; *P* = 0.002) were independent predictors of recovery from dialysis.

**Table 4:** Univariable and multivariable Cox regression analyses for recovery from dialysis within 24 h after CRRT initiation among the survivors (n = 332).

Variables	Univariable		Multivariable	
	HR (95% CI) for renal recovery	*P*	HR (95% CI) for renal recovery	*P*
Demographics				
Age (per 1 year increase)	0.74 (0.98–1.01)	0.074	1.00 (0.98–1.01)	0.845
Sex, male	1.25 (0.89–1.76)	0.191	1.19 (0.84–1.69)	0.337
Baseline body weight (per 1.0 kg increase)	1.00 (0.99–1.02)	0.893		
Comorbid disease				
Chronic kidney disease	0.27 (0.15–0.50)	<0.001	0.31 (0.16–0.62)	0.001
Hypertension	0.87 (0.63–1.20)	0.392		
Diabetes mellitus	0.86 (0.61–1.21)	0.384		
Chronic obstructive pulmonary disease	0.59 (0.30–1.16)	0.127		
Liver cirrhosis	0.83 (0.49–1.42)	0.496		
Congestive heart failure	0.67 (0.37–1.20)	0.175		
Solid cancer	0.84 (0.54–1.32)	0.454		
Hematologic cancer	0.97 (0.48–1.99)	0.941		
Infection source				
Respiratory	0.82 (0.58–1.15)	0.250		
Gastrointestinal	0.99 (0.70–1.41)	0.958		
Urinary tract	1.28 (0.91–1.80)	0.164		
Soft tissue	0.44 (0.11–1.79)	0.254		
Unknown	1.49 (0.66–3.37)	0.343		
Severity of illness				
SOFA score (per 1 point increase)	0.87 (0.85–0.90)	<0.001	0.95 (0.92–0.98)	0.002
APACHE II score (per 1 point increase)	0.91 (0.88–0.94)	<0.001	0.94 (0.91–0.97)	<0.001
Vasopressors use	0.51 (0.35–0.94)	0.001		
Ventilator dependency	0.50 (0.33–0.76)	0.001		
Findings at CRRT initiation				
Mean arterial pressure (per 1.0 mmHg increase)	1.00 (0.99–1.01)	0.442		
Fever or hypothermia	1.02 (0.73–1.43)	0.891		
Heart rate (per 1.0 beat/minute increase)	1.00 (0.99–1.01)	0.829		
Oliguria for 6 hours before CRRT (< 0.5 ml/kg/h)	0.28 (0.16–0.47)	<0.001	0.42 (0.24–0.72)	0.002
Serum creatinine (per 1.0 mg/dl increase)	0.88 (0.74–1.04)	0.131		
Serum blood urea nitrogen (per 1.0 mg/dl increase)	1.00 (0.99–1.00)	0.723		
Sodium (per 1.0 meq/l increase)	0.99 (0.97–1.02)	0.569		
Potassium (per 1.0 meq/l increase)	0.88 (0.74–1.04)	0.125		
Leukocyte count (per 1000/mm^3^ increase)	1.01 (0.99–1.03)	0.243		
Hemoglobin (per 1.0 g/dl increase)	1.05 (0.97–1.13)	0.221		
Platelet count (per 1000/mm^3^ increase)	1.00 (1.00–1.00)	0.672		
Total bilirubin (per 1.0 mg/dl increase)	0.95 (0.88–1.03)	0.233		
Albumin (per 1.0 g/dl increase)	1.14 (0.84–1.55)	0.404		
PT(INR) (per 1.0 increase)	0.89 (0.67–1.18)	0.406		
CRP (per 1.0 mg/dl increase)	0.99 (0.98–1.00)	0.149		
pH (per 0.1 increase)	1.16 (0.21–6.50)	0.869		
Lactate (per 1.0 mmol/l increase)	0.96 (0.91–1.01)	0.077	1.05 (1.00–1.11)	0.070
Interval time from AKI diagnosis to CRRT initiation (per 1 day increase)	0.91 (0.76–1.09)	0.288		
Prescribed CRRT dose (per 1 ml/kg/h increase)	1.02 (0.96–1.08)	0.542		
PCT concentration at CRRT initiation (per 1 ng/ml increase ng/ml)	0.98 (0.96–1.00)	0.016	1.00 (0.98–1.01)	0.688
% PCT decrease^a^ (per 10% decrease)	1.28 (1.21–1.36)	<0.001	1.22 (1.15–1.29)	<0.001

Values are expressed as median (interquartile range) or percentage (%). AKI, acute kidney injury; APACHE II, Acute Physiology and Chronic Health Evaluation II; COPD, chronic obstructive pulmonary disease; CI, confidence interval; CRP, C-reactive protein; CRRT, continuous renal replacement therapy; HR, hazard ratio; PCT, procalcitonin; PT (INR), prothrombin time (international normalized ratio); SOFA, Sequential Organ Failure Assessment

^a^% PCT decrease = [(PCT0-PCT72h)/PCT0] × 100. The % PCT decrease was positive with decreasing concentration and negative with increasing concentration.

Next, an association between the % PCT decrease and recovery from dialysis was investigated in the predefined subgroups (Fig 2B). According to the median SOFA score and time from AKI diagnosis to CRRT initiation, all the survivors were categorized into the high SOFA group (> 11 points) and low SOFA group (≤ 11 points) or the late CRRT group (> 0.3 days) and early CRRT group (≤ 0.3 days). The % PCT decrease was an independent predictor of the recovery from dialysis within 28 days after CRRT initiation across the predefined subgroups.

### Performance of % PCT decrease for predicting the survival and the recovery from dialysis

**Fig 3:**
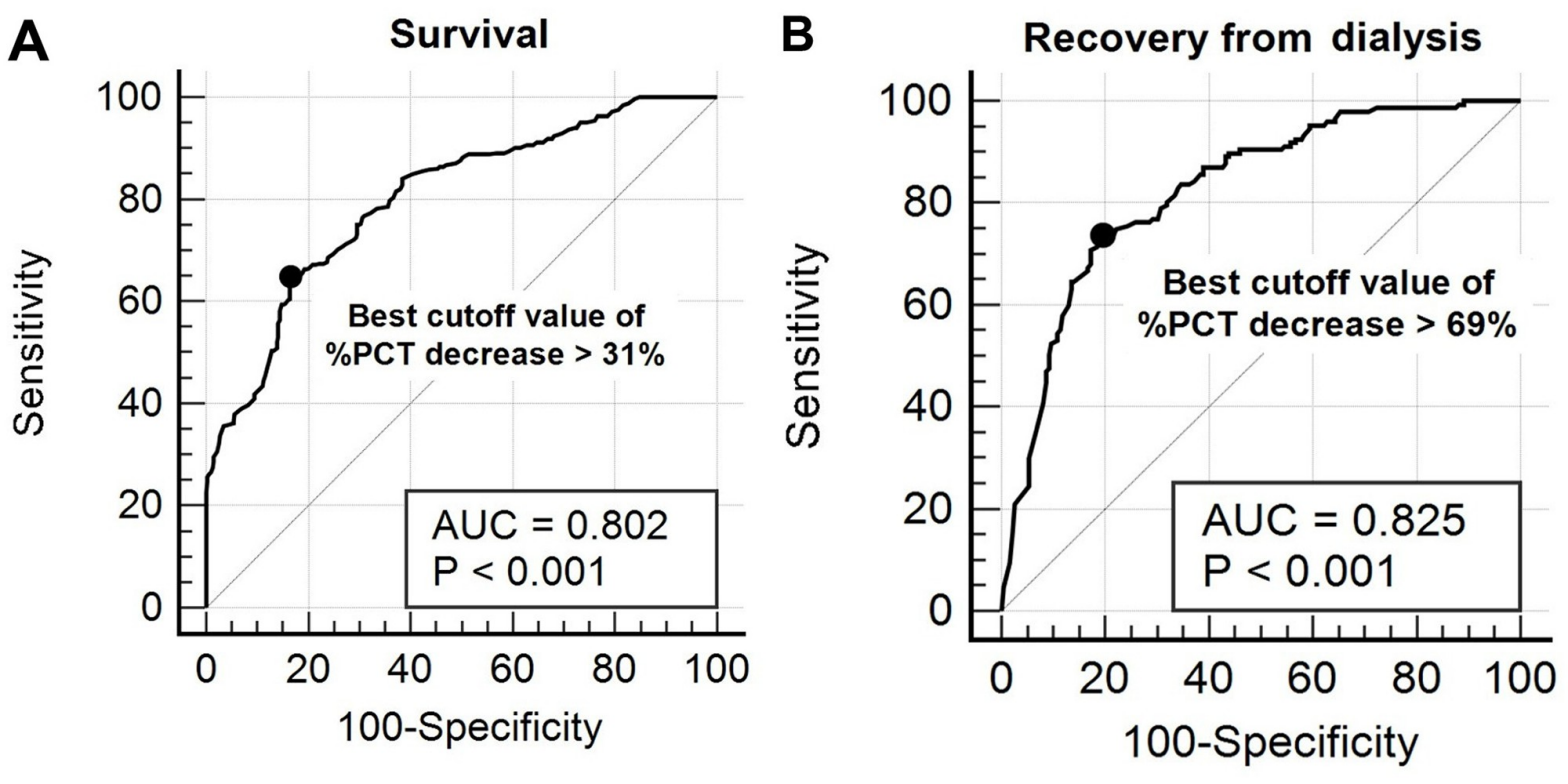
Receiver-operating characteristic curves of % PCT decrease for predicting survival (A) and recovery from dialysis (B) within 28 days after CRRT initiation in patients with SIAKI receiving CRRT. For all the participants (n = 649), the best cut-off value of % PCT decrease for predicting survival was > 31%, with an associated sensitivity of 64.8% and specificity of 83.6% (AUC: 0.802, 95% CI: 0.769–0.832, *P* < 0.001, Youden index: 0.48). In the survivors (n = 332), the best cut-off of % PCT decrease for predicting the recovery from dialysis was > 69%, with an associated sensitivity of 73.5% and specificity of 80.5% (AUC: 0.825, 95% CI: 0.780–0.864, *P* < 0.001, Youden index: 0.54). AKI, acute kidney injury; AUC, area under the curve; CI, confidence interval; CRRT, continuous renal replacement therapy; PCT, procalcitonin; SIAKI, sepsis-induced acute kidney injury.

A ROC analysis was performed to investigate the diagnostic power of % PCT decrease for predicting survival (Fig 3A) and recovery from dialysis (Fig 3B) within 28 days after CRRT initiation. For all the participants, the best cut-off value of % PCT decrease for predicting survival was > 31%, with an associated sensitivity of 64.8% and specificity of 83.6% (AUC, 0.802; 95% CI, 0.769–0.832; *P* < 0.001; Youden index, 0.48). For the survivors, the best cut-off of % PCT decrease for predicting recovery from dialysis was > 69%, with an associated sensitivity of 73.5% and specificity of 80.5% (AUC, 0.825; 95% CI, 0.780–0.864; *P* < 0.001; Youden index, 0.54).

**Fig 4:**
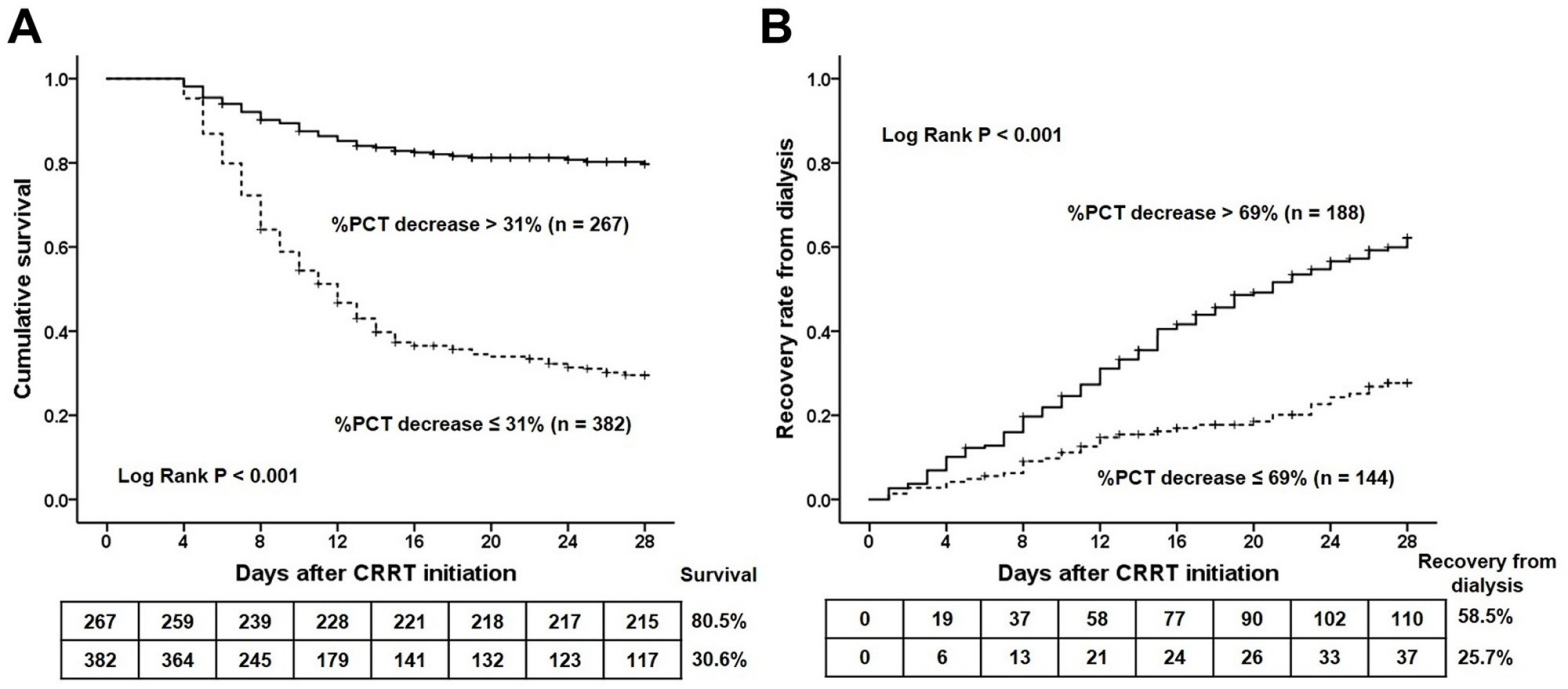
Probability of survival (A) and recovery from dialysis (B) according to the best cut-off value of % PCT decrease in patients with SIAKI receiving CRRT. In all the study subjects (n = 649), patients with a % PCT decrease > 31% showed a significant increase in the survival compared with those with % PCT decrease ≤ 31% (28-day survival: 80.5 vs. 30.6%, *P* < 0.001). In survivors (n = 332), patients with a % PCT decrease > 69% showed a significant higher rate of recovery from dialysis than those with a % PCT decrease ≤ 69% (28-day recovery rate from dialysis: 58.5% vs. 25.7%, *P* < 0.001). CRRT, continuous renal replacement therapy; PCT, procalcitonin; SIAKI, sepsis-induced acute kidney injury.

Next, we divided all the participants into those with % PCT decrease > 31% and ≤ 31%, according to the best cut-off value of % PCT decrease for predicting survival. Patients with a % PCT decrease > 31% showed a significant increase in survival compared to those with a % PCT decrease ≤ 31% (28-day survival: 80.5 vs. 30.6%, *P* < 0.001) (Fig 4A). The survivors were divided into those with a % PCT decrease > 69% and ≤ 69% according to the best cut-off value of the % PCT decrease for predicting recovery from dialysis. Survivors with a % PCT decrease > 69% showed a significantly higher rate of recovery from dialysis than those with a % PCT decrease ≤ 69% (28-day recovery rate from dialysis: 58.5% vs. 25.7%, *P* < 0.001) (Fig 4B).

## Discussion

In the current retrospective study, we found that dynamic changes in the PCT levels over 72 hours after CRRT initiation predicted survival and recovery from dialysis at 28 days in patients with SIAKI receiving CRRT. This finding was independent of the initial disease severity assessed by commonly used clinical risk scores, including SOFA or APACHE II. The predictive value of a single PCT level at CRRT initiation is poor. We demonstrated the best cut-off value of % PCT decrease for predicting survival and recovery from dialysis, which suggested that a decrease in % PCT could be a promising biomarker for predicting the prognosis in these patients.

Accurate assessments of disease severity and prediction of the clinical course helps patients, families, and caregivers to set reasonable expectations about the illness [11]. Accurate risk stratification for prognosis is also required for the proper application of healthcare resources and treatment modalities [11]. For the management of patients with SIAKI receiving CRRT, which is associated with a high risk of survival and recovery from dialysis [6, 7], accurate risk stratification for prognosis is particularly important. Established clinical risk scores, such as SOFA or APACHE, have been used for the risk stratification of patients with sepsis [11]. However, clinical risk scores are somewhat constrained by practical concerns and are only validated when admission values are employed. Further, the efficacy of monitoring these scores in sepsis is not well established [11]. Owing to differences in the patient groups with for which the scores were produced and implemented, the scores may potentially suffer from miscalibration and as a result, have only modest operational features [11]. Thus, there is an interest in the discovery of biomarkers that are rapidly measurable, respond immediately to clinical recovery, and provide relevant, reliable, and real-time information in patients with sepsis [20].

PCT has attracted considerable interest as a sepsis biomarker for predicting a higher risk and severity of bacterial infections [17]. PCT has also been reported to be associated with an increased risk of bacteremia as confirmed by bacteria cultures [17]. Although the actual function of PCT in the host defense system is unknown, this peptide has numerous effects on the immune system, including a decrease in neutrophil phagocytic and candidacidal activity as well as an increase in intracellular calcium ions, all of which aid the host response [17]. The present study demonstrated that the % PCT decrease over 72 hours after CRRT initiation has a prognostic value for predicting survival and recovery from dialysis at 28 days in patients with SIAKI receiving CRRT. In terms of biomarkers, a decrease in the % PCT is more easily assessable than clinical risk scores, such as SOFA or APACHE. Thus, the results of the present study underline that a % PCT decrease provides an easy, economic, and fast approach for predicting prognosis in patients with SIAKI receiving CRRT.

Most previous studies on the prognostic value of PCT have included patients with sepsis, including severe sepsis and septic shock [14], whereas we included only patients with SIAKI receiving CRRT in the present study. To the best of our knowledge, the present study is the first to investigate the association between PCT levels and survival or recovery from dialysis in patients with SIAKI receiving CRRT. Previous studies on the prognostic value of PCT in predicting survival in patients with sepsis have produced conflicting results. In a recent meta-analysis that included 16 studies with 3126 patients with sepsis, an initial single PCT level was associated with a higher risk of death (pooled relative risk: 2.60, 95% CI: 2.05–3.30) [14]. In the same meta-analysis, including nine studies with 868 patients with sepsis, PCT non-clearance was a prognostic factor for death in patients with sepsis (pooled relative risk: 3.05, 95% CI: 2.35–3.95) [14]. In the present study, the multivariable analysis demonstrated that the % PCT decrease over 72 hours after CRRT initiation was an independent predictor of the 28-day survival in patients with SIAKI receiving CRRT (per 10% decrease, HR: 0.91, 95% CI: 0.89–0.93; *P* < 0.001). A single PCT level at CRRT initiation was associated with the 28-day survival in the univariable analysis, but this association was not statistically significant in the multivariable analysis. However, the reasons for these findings remain unclear. The initial absolute peak of PCT in patients with sepsis occurs early on—it reaches a plateau value at 6–24 hours and has a half-life of approximately 24–35 hours [21]. Thus, the measurement of PCT at CRRT initiation may be of limited value due to the variability of PCT secretion at different phases of sepsis and different times of relapse between the onset of sepsis and CRRT initiation. Therefore, it is assumed that a single PCT level at CRRT initiation was not associated with a poor prognosis in our study subjects. In contrast to a single PCT level, a dynamic approach to assessing PCT levels can capture the progression of sepsis and reflect the effectiveness of sepsis management. Therefore, we assumed that dynamic changes in the PCT levels over 72 hours after CRRT initiation were associated with poor prognoses in the participants of our study. Although the reason for these findings should be clarified in further studies, the results of our study suggest that dynamic changes in the PCT level over 72 hours after CRRT initiation, rather than a single PCT level at CRRT initiation, predicts 28-day survival in patients with SIAKI receiving CRRT.

Regarding the association between PCT and AKI, previous studies suggested that initial PCT concentration is a potential biomarker for predicting AKI occurrence in various clinical settings, including patients with sepsis [15]. However, to date, the prognostic role of PCT in predicting renal function recovery in patients with established AKI remains unknown. In the present study, the % PCT decreased over 72 hours after CRRT initiation; however, the initial PCT level at CRRT initiation was an independent predictor of recovery from dialysis in patients with SIAKI who survived after CRRT initiation. The present study demonstrated that CKD, SOFA scores, APACHE II scores, and oliguria, which have been reported to be predictors of recovery from dialysis in patients receiving CRRT [22], were independent predictors of recovery from dialysis in patients with SIAKI receiving CRRT, in addition to the decrease in the % PCT. All these findings suggest that in addition to the traditional predictors, the % PCT dynamic change over 72 hours after CRRT initiation rather than a single PCT level at CRRT initiation predicts the recovery from dialysis in patients with SIAKI receiving CRRT.

The cut-off value of the % PCT decrease for predicting prognosis is of interest currently. Previous studies have demonstrated that % PCT decreases within a range of 30–70% within 2–7 days after the initial PCT measurement predicted survival in patients with sepsis [14]. The present study showed that the best cut-off value of % PCT decrease over 72 hours after CRRT initiation for predicting survival in patients with SIAKI receiving CRRT was > 31% (AUC: 0.802, *P* < 0.001), with an associated sensitivity of 64.8% and specificity of 83.6%. Patients with a % PCT decrease > 31% showed a significant increase in survival compared to those with a % PCT decrease ≤ 31% (28-day survival: 80.5 vs. 30.6%, *P* < 0.001). Furthermore, for the first time, to the best of our knowledge, the present study showed that the best cut-off of % PCT decrease for predicting recovery from dialysis in survivors was > 69% (AUC: 0.825, 95% CI: 0.780–0.864, *P* < 0.001) with an associated sensitivity of 73.5% and specificity of 80.5%. Survivors with a % PCT decrease > 69% showed a significantly higher rate of recovery from dialysis than those with a % PCT decrease ≤ 69% (28-day recovery rate from dialysis: 58.5% vs. 25.7%, *P* < 0.001). From these findings, we suggest that the serial monitoring of PCT concentration over 72 hours after CRRT initiation could help guide physicians to identify patients who are at risk of death and are dialysis-dependent in patients with SIAKI receiving CRRT, and prompt physicians to evaluate the appropriateness and adequacy of early management in these patients.

The present study has several strengths. First, although the subjects of the present study were limited to patients with SIAKI receiving CRRT, it included a relatively large number of patients (n = 649) compared with previous studies that examined the prognostic value of PCT decrease in patients with sepsis (sample size, n = 27–242) [14]. Second, our multivariable model included adjustments for important confounding variables that are reported to impact survival and recovery from dialysis in patients with AKI receiving CRRT, such as oliguria, SOFA scores, APACHE II scores, and interval time from AKI diagnosis to CRRT initiation. All these findings provide more substantial evidence of the association between PCT and survival or recovery from dialysis at 28 days in patients with SIAKI receiving CRRT.

Despite its strengths, our study had some limitations. First, owing to its retrospective design, it was difficult to establish a causal relationship between the % PCT decrease and survival or recovery from dialysis and to draw conclusions about the clinical effects of serial PCT monitoring in our study subjects. Thus, the results of the present study should be validated in future prospective and interventional studies, to verify whether serial PCT monitoring can improve the clinical decisions and outcomes. Second, we included a specific subset of critically ill patients, namely, those with SIAKI who received CRRT. Thus, a selection bias cannot be avoided, and the results of our study might not be generalizable to other populations of critically ill patients with AKI. Third, the actual delivered dose of CRRT, which may affect PCT removal, was not investigated in this study. PCT has a molecular weight of 13.5 kDa [23]. Although it remains controversial whether the removal of inflammatory cytokines, including PCT, by CRRT improves the outcome in patients with sepsis, PCT has been reported to be detected in the ultrafiltrate of patients receiving CRRT and eliminated by convection [23, 24]. Although the prescribed dose of CRRT did not differ between survivors and non-survivors in the present study (37.6 [35.4–39.9] vs. 37.5 [35.0–40.0], *P* = 0.514), we cannot rule out the possibility that the actual delivered dose of CRRT could affect PCT removal and patient outcomes.

## Conclusions

The present study showed that a decrease in % PCT was an independent predictor of survival and recovery from dialysis in patients with SIAKI receiving CRRT. We also demonstrated the best cut-off value of % PCT decrease for predicting survival and recovery from dialysis in these patients. We showed that the % PCT decrease, which can be obtained by a simple calculation, could assist physicians in identifying patients with a high-risk profile and initiating timely intervention in patients with SIAKI receiving CRRT.
